# Epidermal Choristoma of the Tongue: A Potential Diagnostic Pitfall

**DOI:** 10.1155/crid/1327746

**Published:** 2026-09-24

**Authors:** Rana Saud Alshagroud, Bader Mutrik Aldawsari, Hassan Ibrahim Khormi, Yara Ibrahim Alromaih, Azizah Bin Mubayrik

**Affiliations:** ^1^ Department of Oral Medicine and Diagnostic Sciences, College of Dentistry, King Saud University, Riyadh, Saudi Arabia, ksu.edu.sa; ^2^ Maxillofacial Surgery and Rehabilitation Department, King Fahad Medical City, Riyadh, Saudi Arabia, kfmc.med.sa

## Abstract

We report a rare case of epidermal choristoma of the tongue in a 52‐year‐old female, presenting as an asymptomatic brown‐to‐black macule clinically resembling a melanotic macule. Excisional biopsy revealed abrupt transition from oral mucosal epithelium to epidermis‐like stratified squamous epithelium with hyperkeratosis, hypergranulosis, basal cell melanosis, and mild melanocytic hyperplasia, along with sebaceous adnexal structures in the lamina propria, confirming the diagnosis. Epidermal choristoma is an exceptionally rare developmental lesion of ectodermal origin, with nine oral cases reported to date. Due to its nonspecific clinical appearance, it may be mistaken for benign melanotic lesions or early mucosal melanoma. Complete surgical excision is curative, with no recurrence observed at 15‐month follow‐up in this case. This report highlights the importance of histopathologic evaluation of pigmented tongue lesions to avoid misdiagnosis and ensure appropriate management.

## 1. Introduction

Choristoma is defined as a cohesive, tumor‐like mass composed of normal, mature tissue located in an abnormal site [1]. It arises due to failures in the normal course of embryonic development, particularly during the formation of the neural tube, which can lead to a variety of developmental defects as the head and neck regions evolve [1]. In the oral cavity, choristomas are classified based on the type of tissue they contain and include several variants such as salivary gland choristomas, cartilaginous choristomas, osseous choristomas, lingual thyroid choristomas, lingual sebaceous choristomas, glial choristomas, and gastric/respiratory mucosal choristomas [2]. Among these, epidermal choristoma of the oral cavity represents an exceptionally rare, benign developmental lesion that is frequently misdiagnosed clinically due to its resemblance to melanotic macules, typically presenting as brown plaques or macules on the tongue [3]. It is characterized microscopically by the replacement of normal oral mucosal epithelium with epidermis‐like epithelium [3]. Only a limited number of cases have been reported in the literature; herein, we present an additional case of this exceptionally rare lesion on the tongue, clinically mimicking a melanotic macule.

## 2. Case Presentation

**Figure 1:**
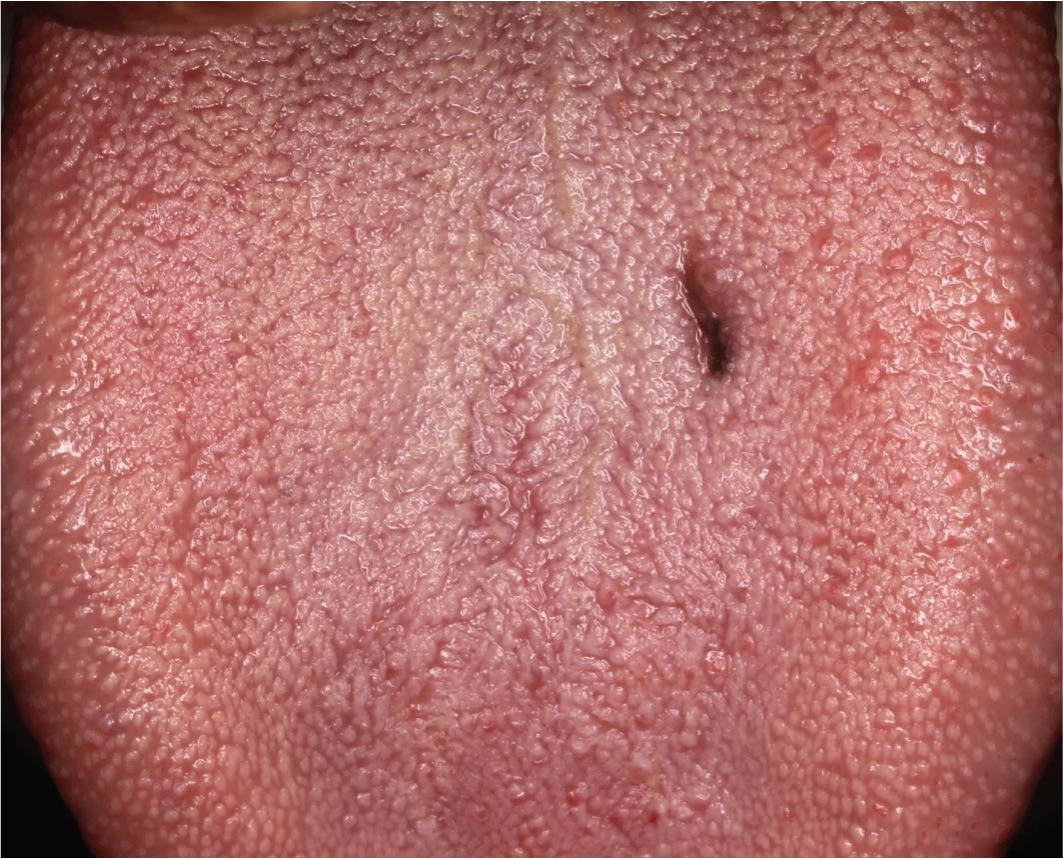
A linear brown‐to‐black pigmented lesion measuring 0.4 × 0.1 cm on the dorsum of the tongue.

A 52‐year‐old female presented to the oral medicine clinic with a pigmented lesion on the tongue, initially noticed by her dentist 3 months prior; however, the exact duration of the lesion remains unknown. Her medical history included controlled hypertension, diabetes mellitus, and hyperlipidemia. Her current medications were metformin 750 mg, perindopril 5 mg, and atorvastatin 20 mg. There was no history of tobacco use. Clinical examination revealed a linear brown‐to‐black pigmented lesion measuring 0.4 × 0.1 cm on the dorsum of the tongue (Figure 1). The lesion was asymptomatic, and the remainder of the clinical examination was unremarkable. Based on clinical findings, a provisional diagnosis of melanotic macule was made. Written informed consent was obtained from the patient, and an excisional biopsy was performed under local anesthesia.

**Figure 2:**
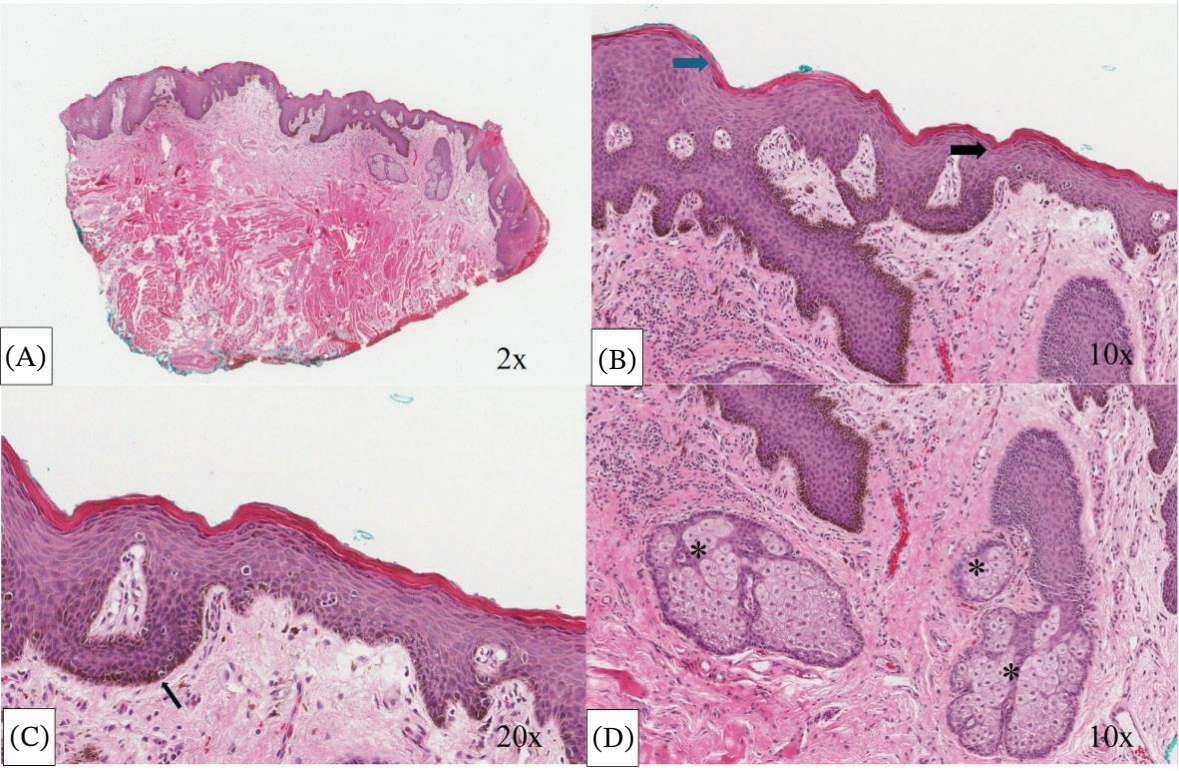
(A) A low‐power magnification shows a wedge of lingual mucosa surfaced by stratified squamous epithelium. (B) A medium‐power magnification of the surface epithelium showing parakeratinization (blue arrow) with an abrupt transition to orthokeratinized epithelium demonstrating hypergranulosis (black arrow). (C) A high‐power magnification showing basal cell melanosis and mild melanocytic hyperplasia (thin arrow). (D) Sebaceous glands identified in the superficial lamina propria at a medium power magnification (∗).

Microscopic examination revealed a wedge of lingual mucosa surfaced by stratified squamous epithelium of variable thickness. The epithelium exhibited parakeratinization at the periphery with an abrupt transition toward the center to orthokeratinized epithelium exhibiting hyperkeratosis, hypergranulosis, basal cell melanosis, mild melanocytic hyperplasia, and basal cell hyperplasia. The rete ridges are in variable shapes ranging from inconspicuous or short to elongated and interconnecting. Additionally, adnexal structures, mainly sebaceous glands, composed of lobules bordered by basophilic basal cells with central large, vacuolated cells “sebocytes” characterized by foamy cytoplasm and small nuclei, were identified in the superficial lamina propria. The deeper lamina propria is composed of mainly lingual musculature with variable vascularity. (Figure 2A–D). These histopathological findings were consistent with a diagnosis of epidermal choristoma. The healing process was uneventful, with no evidence of recurrence observed after a 15‐month follow‐up period.

## 3. Discussion

Epidermal choristoma is a rare, benign developmental overgrowth that typically presents as a pigmented macule or papule on the tongue, often raising clinical concern for a melanocytic lesion [3]. The etiology of epidermal choristomas remains uncertain, despite various proposed theories. It is widely believed that these lesions are developmental in origin, arising from disruptions in normal embryonic development [3, 4]. These disruptions may lead to ectodermal sequestration within the oral epithelium during embryogenesis [3, 4]. Their development could involve the formation of dermal appendages from ectodermal epithelial remnants or may result from the spontaneous marsupialization of an undetected submucosal dermoid cyst [3, 4]. Azorín et al. [5] first described a case involving a 1‐month‐old male who presented with a pigmented macule on the tongue, and they subsequently proposed the term “epidermal choristoma.” To date, nine cases of epidermal choristoma have been reported in the literature, in addition to the present case. Except for one previously reported female case, all documented patients were male, making the current case the second known occurrence in a female. The age range of reported cases was ranging from 1 month to 56 years. The most frequent clinical presentation was a brown‐ to black‐pigmented macule, which could be solitary or multiple, with color varying from light to dark brown, and measuring 3–11 mm in diameter, typically located on the dorsum of the tongue (Table 1) [3–9]. Histologically, it shows an abrupt transition from normal oral mucosal epithelium to epidermis‐like stratified squamous epithelium exhibiting hyperorthokeratosis, hypergranulosis, and melanosis of the basal cells and sometimes associated with melanocytes hyperplasia with underlying cutaneous adnexal structures such as sebaceous glands, hair follicles, and occasionally sweat glands [3]. In contrast to the predominant clinical presentation, one patient was reported with a polypoid lesion on the upper gingiva, another exhibited a white‐brown plaque on the buccal mucosa, and a third presented with a deep midline tongue mass [3, 6, 9]. The gingival polypoid lesion may represent a distinct pathological entity, possibly a hairy polyp, based on its unique morphology and clinical features [3]. Another atypical case, described by Lindquist et al. [9], presented as deep midline tongue mass. Histopathological analysis revealed keratinizing squamous epithelium with acanthosis and hypergranulosis, accompanied by abundant sebaceous gland proliferation, apocrine glands, occasional hair follicles, and mature adipose tissue [9]. Notably, there was an absence of both melanocyte hyperplasia and basal cell melanosis within the epithelium [9]. These findings suggest that this case may also represent a separate entity due to its distinctive clinical and histological characteristics [9]. Various causes of oral pigmentation have been reported and can usually be distinguished through a careful clinical history, thorough examination, and histopathologic evaluation [9]. The clinical differential diagnosis for brown‐pigmented oral lesions is broad and can be systematically categorized according to the distribution of the lesions as either focal (localized) or multifocal/generalized [10]. Focal pigmented lesions include oral melanotic macule, congenital melanocytic macule, melanocytic nevus, oral melanoacanthoma, and melanoma [10]. Developmental or ectopic lesions, such as epidermal choristoma, may also present as focal pigmentation, particularly on the dorsum of the tongue, as observed in the present case. In contrast, multifocal or generalized pigmentation may occur in physiologic (ethnic) pigmentation, smoker′s melanosis, postinflammatory hyperpigmentation, and medication‐induced pigmentation (e.g., antimalarials, chemotherapeutic agents, zidovudine, ketoconazole, and estrogens) [10]. Systemic and syndromic disorders including Addison disease, Peutz–Jeghers syndrome, McCune–Albright syndrome, and neurofibromatosis can also manifest as multifocal or diffuse oral pigmentation [10]. Although epidermal choristoma most commonly presents as a solitary focal lesion, rare presentations with multifocal oral pigmentation have also been described [8]. In the present case, the patient exhibited no symptoms suggestive of systemic involvement, and there was no evidence of multifocal or extraoral pigmentation. Additionally, she was not taking any medications known to induce oral pigmentation. Based on the clinical presentation, an oral melanotic macule was favored as the provisional diagnosis. An excisional biopsy was subsequently performed, and histopathological examination confirmed the diagnosis of epidermal choristoma. It is possible that some cases of epidermal choristoma are clinically misinterpreted as melanotic macules or other benign pigmented lesions of the oral mucosa, resulting in underrecognition, as such lesions are often not subjected to biopsy [3]. However, clinicians should remain mindful that any oral pigmented lesion of uncertain origin warrants biopsy and histopathologic examination to exclude oral mucosal melanoma and other melanocytic entities [3]. Although oral melanoma most commonly arises on the palatal mucosa and maxillary gingiva, its occurrence on the tongue or buccal mucosa is uncommon but possible [3]. The histopathological features of epidermal choristoma are very distinctive, and when correlated with the clinical findings, they support a definitive diagnosis [3, 5]. Nevertheless, the histopathologic differential diagnosis includes several lesions with overlapping microscopic features, such as oral melanotic macule, congenital melanotic macule, melanocytic nevus, Fordyce granules, hairy polyp, dermoid cyst, and oral linear epidermal nevus (LEN) [3, 5–7]. Oral melanotic macule and congenital melanotic macule may clinically resemble an epidermal choristoma; however, microscopically they demonstrate increased basal melanin pigmentation without the presence of adnexal structures or epidermal‐type differentiation [10–12]. In contrast, a melanocytic nevus can also mimic epidermal choristoma clinically but histologically shows nests of nevus cells within the epithelium and/or underlying connective tissue [13]. Like the melanocytic macule, it lacks adnexal elements, distinguishing it from epidermal choristoma, which characteristically contains sebaceous glands, hair follicles, and occasionally sweat glands. Fordyce granules are ectopic sebaceous glands located within the oral mucosa [14]. Clinically, they present as multiple, 1–3 mm, yellowish papules, most commonly observed on the posterior buccal mucosa and the vermilion border of the lips, although they may appear at any intraoral site [14]. The lesions are usually bilateral and symmetrical [14]. Histopathologically, they consist of clusters of mature sebaceous lobules situated immediately beneath a nonkeratinized stratified squamous epithelium. These lobules lack associated hair follicles or other adnexal structures [14]. A hairy polyp typically presents clinically as a polypoid mass [15]. Histologically, it contains ectodermal components, including skin adnexal structures such as hair follicles and sebaceous glands; however, it also exhibits mesodermal elements, such as adipose tissue, skeletal muscle, or cartilage, which are absent in epidermal choristoma [15]. A dermoid cyst is a developmental lesion that clinically presents as a dome‐shaped, doughy, painless, yellowish nodule or mass, most commonly located in the midline of the floor of the mouth [16, 17]. Microscopically, it is characterized by a cystic cavity lined by keratinized stratified squamous epithelium and containing skin adnexal structures, including sebaceous glands, hair follicles, and sweat glands, within its cyst wall [16, 17]. It differs from an epidermal choristoma by its cystic architecture. LEN may be considered in the histopathologic differential diagnosis of epidermal choristoma, as both lesions exhibit epidermal‐type stratified squamous epithelium with hyperkeratosis, acanthosis, and adnexal differentiation [18]. LEN represents a hamartomatous proliferation of epidermal and adnexal structures arising within their normal anatomic location, typically following the lines of Blaschko on the skin or appearing at mucocutaneous junctions [18]. It commonly manifests during infancy or early childhood as linear, pigmented, and verrucous papules or plaques that usually occur unilaterally on the skin [18]. LEN may be associated with epidermal nevus syndromes, which can involve neurological, skeletal, or ocular abnormalities [18]. Oral involvement is rare and typically occurs in association with cutaneous lesions, whereas isolated oral presentations without skin manifestations are exceedingly uncommon [18]. The corresponding oral lesions often present as papillary plaques that are extensive and multifocal, generally mucosa‐colored, and arranged in a unilateral or occasionally midline distribution [18]. The lips, tongue, and palatal mucosa are the most frequently affected intraoral sites [18]. LEN is characterized by activating *RAS* mutations [19]. In contrast, epidermal choristoma represents a choristomatous lesion composed of normal skin tissue located at an ectopic intraoral site, such as the tongue [18]. Therefore, although LEN may be considered when histologic sections reveal epidermal hyperplasia with adnexal differentiation, it can be excluded based on its distinct clinical distribution, presentation pattern, and syndromic associations [18]. Recently, ultrasonography, particularly high‐frequency (up to 70 MHz) and color Doppler ultrasound, has been used to diagnose and monitor epidermal choristoma [20]. It provides high‐resolution images, allowing for detailed visualization of submucosal structures such as sebaceous glands [20]. This noninvasive technique is especially useful in children, where biopsy may be complex and risky [20]. Excision of epidermal choristomas is curative, with no reported cases of recurrence [3]. For larger epidermal choristomas, observation may be a reasonable alternative to complete excision if histopathology confirms the diagnosis, particularly to preserve the integrity and function of the tongue [8]. Hughes et al. [8] reported three cases of epidermal choristoma, two of which were associated with other pigmented skin lesions. One patient presented with lumbosacral and right leg dermal melanocytosis consistent with congenital dermal melanocytosis, whereas the other exhibited midline dorsal congenital dermal melanocytosis and three café‐au‐lait macules [8]. This finding is noteworthy and may suggest an underlying developmental or genetic background [8]. Further investigations are warranted to determine whether epidermal choristomas exhibit molecular alterations similar to those described in mosaic hyperpigmentation disorders [21].

**Table 1:** Reported cases of oral epidermal choristoma.

Author	Year	Age	Sex	Country	Size	Clinical Presentation	Treatment	Follow‐up
**Chi et al. (Case 1)^3^**	2010	32 years	Male	United States	1.1 × 0.8 cm	Well‐defined, firm, leathery, brown‐and‐white plaque	Observation	No changes reported (1‐month follow up period)
**Chi et al. (Case 2)^3^**	2010	56 years	Male	United States	0.3 cm in maximum diameter	Single brown pigmented macule	Excision	No recurrence reported (3‐month follow up period)
**Azorín et al.^5^**	2005	1 month	Male	Spain	0.8 × 0.3 cm	Three dark brown macules	Excision	No recurrence reported (9‐month follow up period)
**Yoshioka et al.^6^**	2012	2 months	Male	Japan	1 × 0.6 cm	Pedunculated polypoid mass (clinically: congenital epulis)	Excision	No recurrence reported (1‐year follow up period)
**Curto-Barredo et al.^7^**	2015	1 month	Male	Spain	0.4 × 0.3 cm	Light‐to‐dark brown pigmented macule (mimicked congenital melanotic macule)	Excision	No recurrence reported (9‐month follow up period)
**Hughes et al. (Case 1)^8^**	2021	7 weeks	Male	United Kingdom	1 × 0.5 cm	Three clustered dark brown macules (congenital)	Observation	Stable (3‐year follow up period)
**Hughes et al. (Case 2)^8^**	2021	21 months	Male	United Kingdom	0.3 × 0.3 cm	Clustered dark brown macules	Observation	Stable (15‐month follow up period)
**Hughes et al. (Case 3)^8^**	2021	6 years	Male	United Kingdom	2 × 1.1 cm	Light brown hyperpigmented plaque	Excision	No recurrence reported
**Lindquist et al.^9^**	2017	14 years	Female	United States	1 × 1 cm	Deep dorsal tongue mass	Excision	No recurrence reported (1‐month follow up period)
**Present case**	2025	52 years	Female	Kingdom of Saudi Arabia	0.4 × 0.1 cm	One linear pigmented macule	Excision	No recurrence reported (15‐month follow up period)

## 4. Conclusion

In summary, we report the tenth documented case of epidermal choristoma arising within the oral cavity. This entity represents an exceptionally rare, benign developmental anomaly of ectodermal origin. It most commonly presents as a pigmented macule located on the dorsal surface of the tongue. Given its nonspecific clinical appearance, epidermal choristoma should be included in the differential diagnosis of pigmented lesions of the tongue and distinguished from other pigmented conditions through histopathologic evaluation.

## Funding

No funding was received for this manuscript.

## Conflicts of Interest

The authors declare no conflicts of interest.

## Data Availability

The data that support the findings of this study are available on request from the corresponding author. The data are not publicly available due to privacy or ethical restrictions.
